# Supplementary figures and images for: Initiation and spread of escape waves within animal groups
Source: R Soc Open Sci. 2015 Apr 1;2(4):140355. doi: 10.1098/rsos.140355 (PMC4448869; doi:10.1098/rsos.140355)

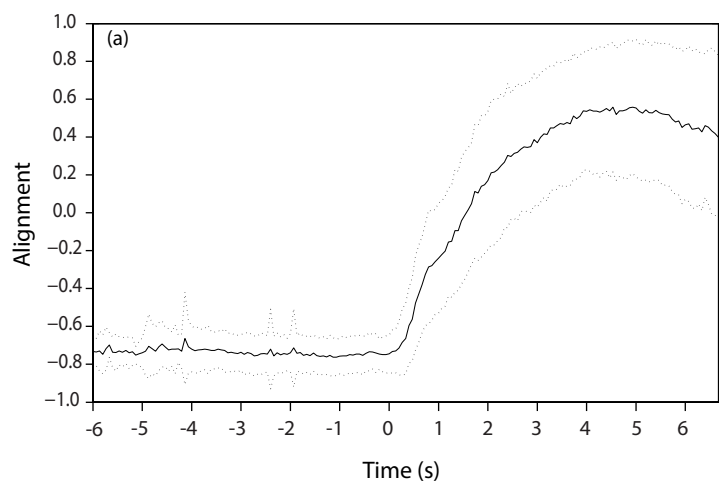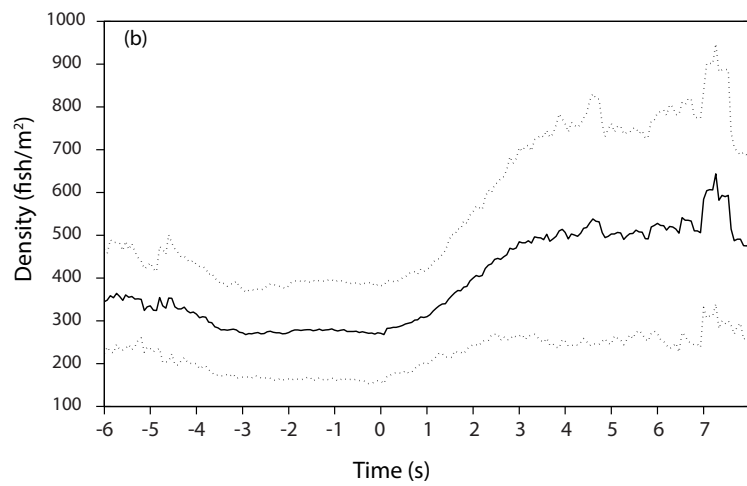

Supplement: Figure S1. Collective alignment and density of fish before and after presentation of the stimulus. (a) Collective alignment and (b) density of fish before (negative seconds) and after (positive seconds) the stimulus entered the arena. The shoals show an increase in alignment and density following th [file rsos140355supp1.pdf]

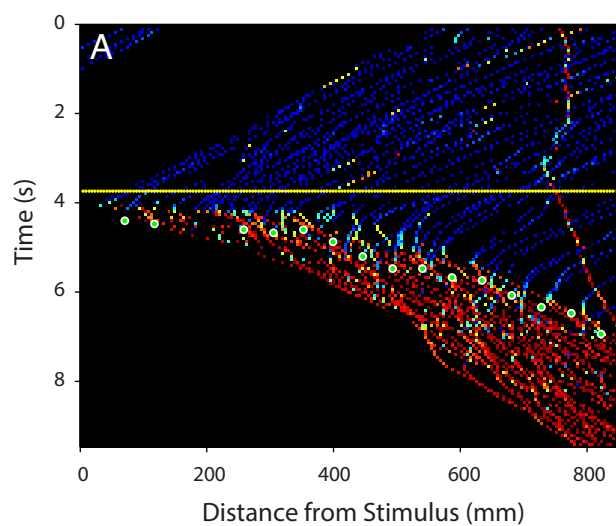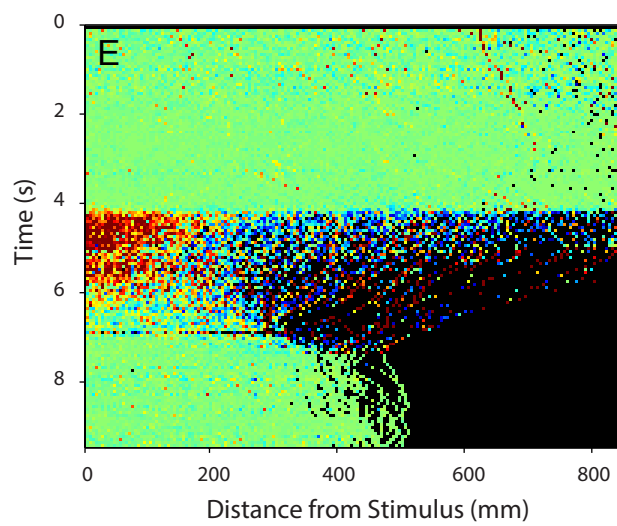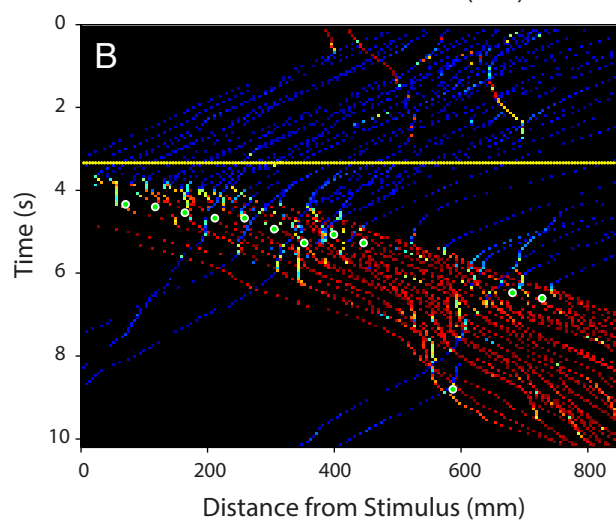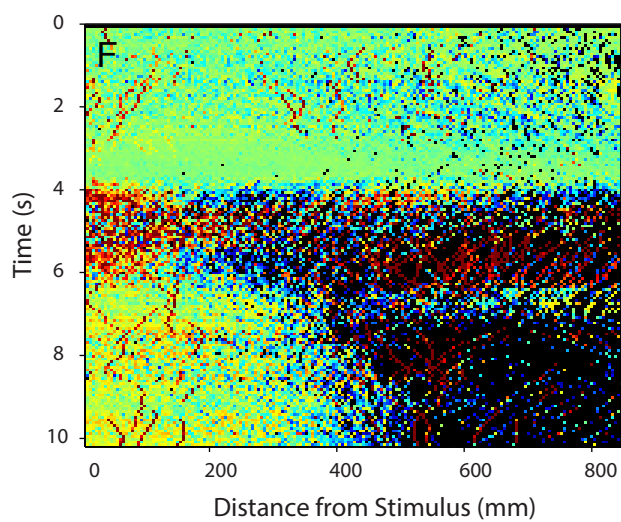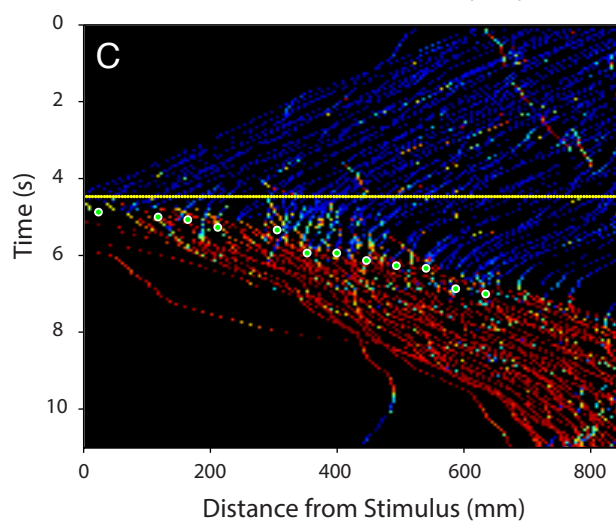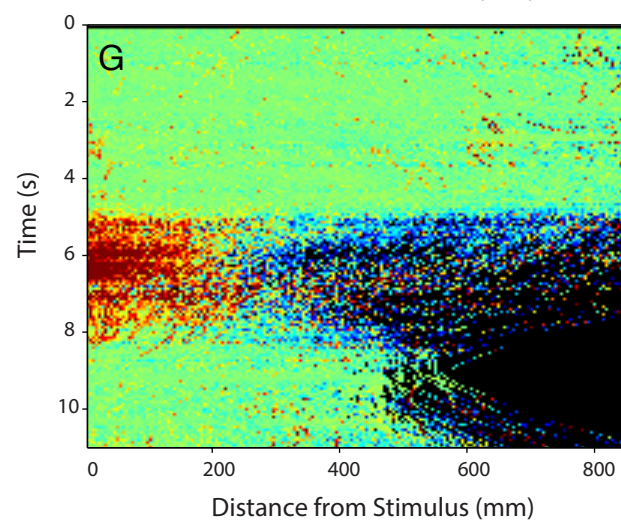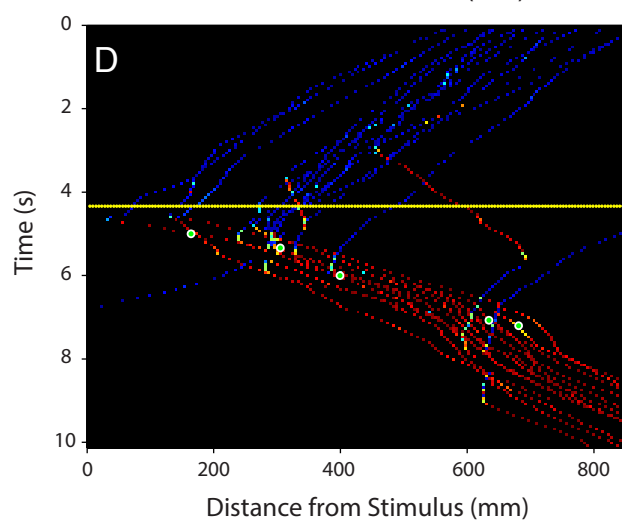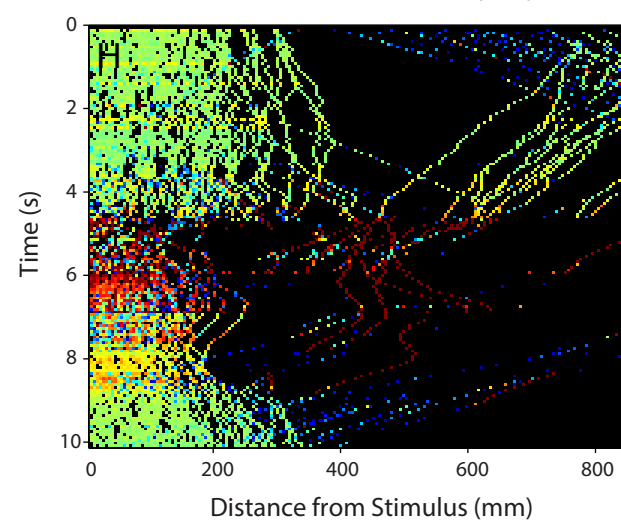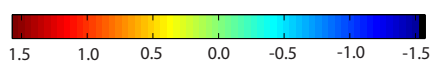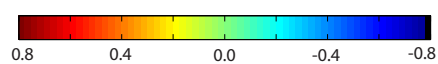

Supplement: Figure S2. Examples of the dynamics of information transfer and spatial velocity fluctuations in the experimental trials. Trials with (a, e) 63 fish; (b, f) 45 fish; (c, g) 98, (d, h) 16. (a-d) Each line represents a one dimensional view of the arena at a given frame where each individual’s angular  [file rsos140355supp2.pdf]

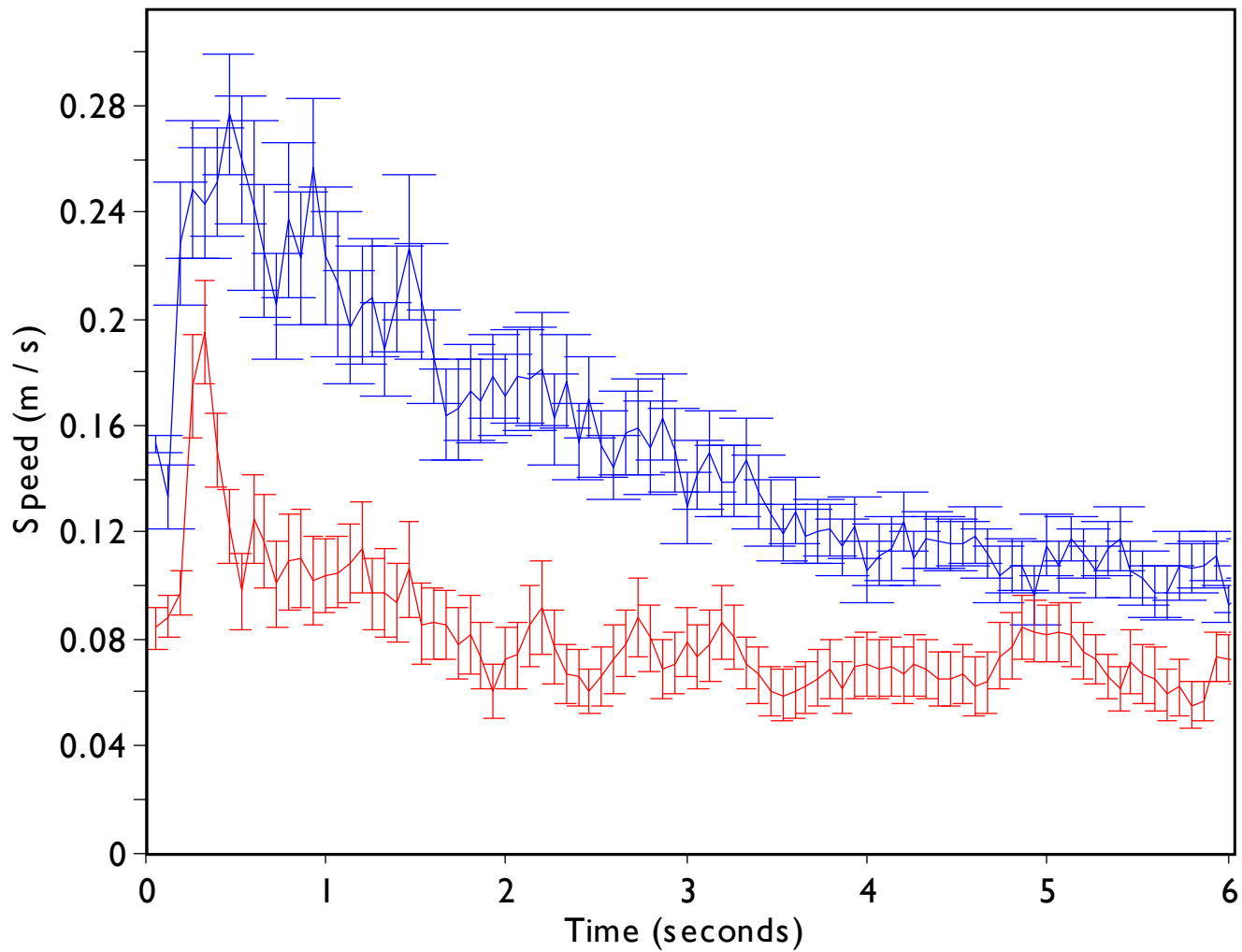

Supplement: Figure S3. Speed profiles of individual fish reacting to the stimulus. The average speed of the first responding individual in groups (blue line) and when individuals were trialled on their own (red line) after the stimulus entered the arena at 0 seconds. Fish in both contexts show a rapid increase  [file rsos140355supp3.pdf]

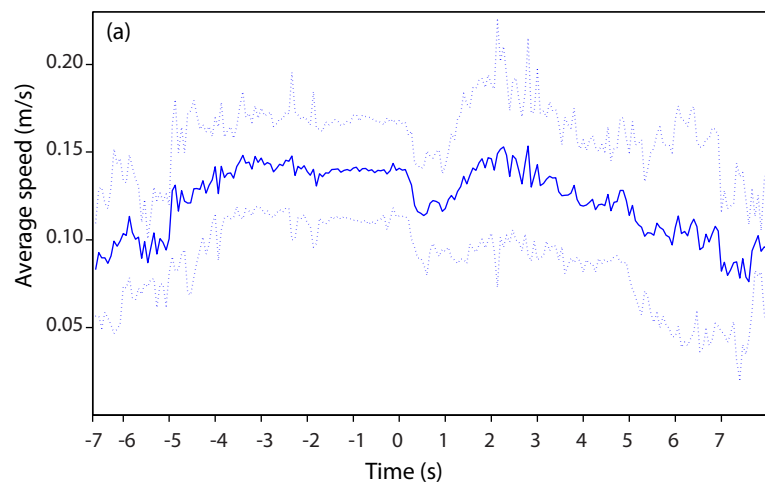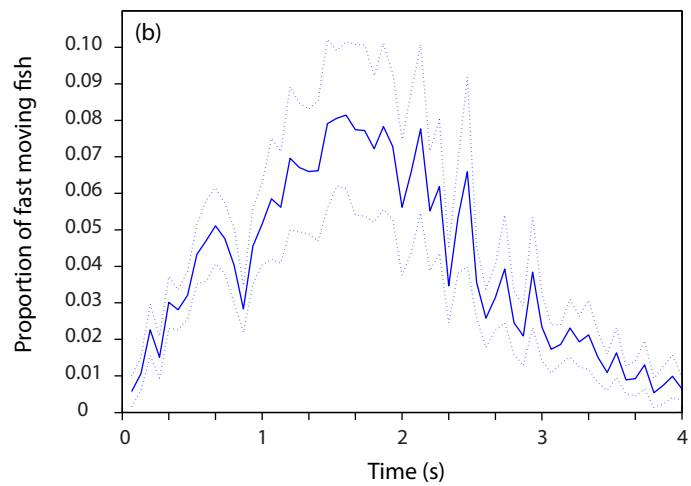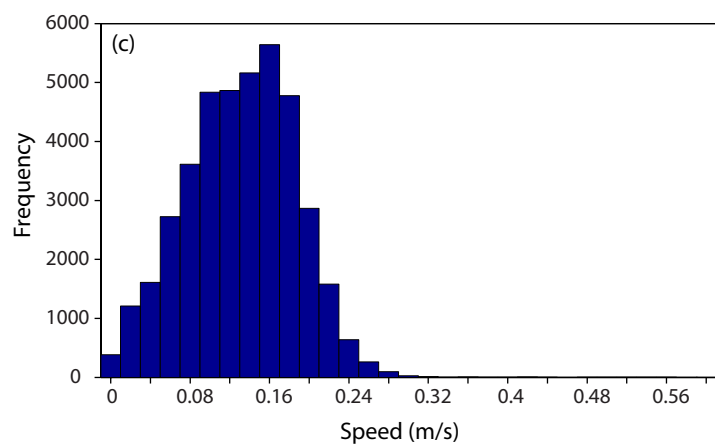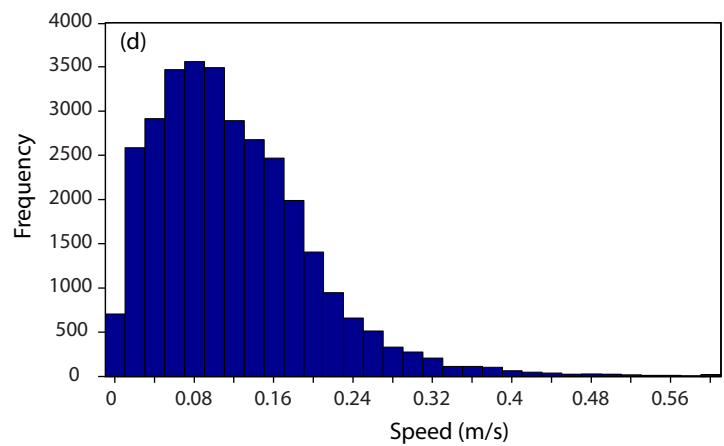

Supplement: Figure S4. Distribution of fish speeds before and after the presentation of the stimulus. (a) Mean speed of all fish in trials (± 1 SD) before (negative seconds) and after (positive seconds) the stimulus enters the arena at 0 seconds. Between −4 to 0 seconds the fish reach an average cruise speed of [file rsos140355supp4.pdf]

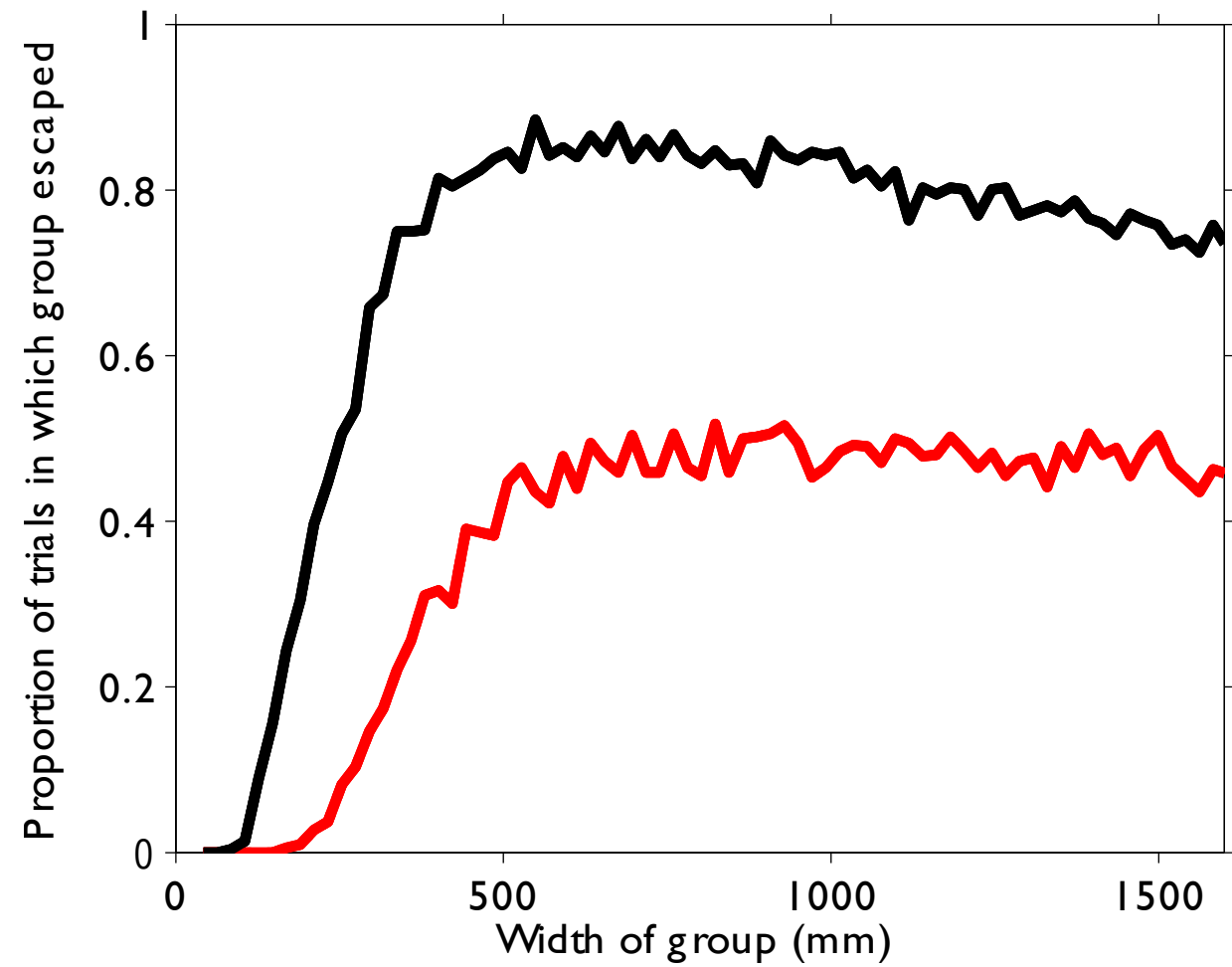

Supplement: Figure S5. The role of speed change in determining whether a group changes direction. Results of 1024 simulation runs of 80 individuals, in which 8 are informed. The dotted line shows the proportion of runs in which the group changed direction as a function of group width when informed and uninforme [file rsos140355supp5.pdf]

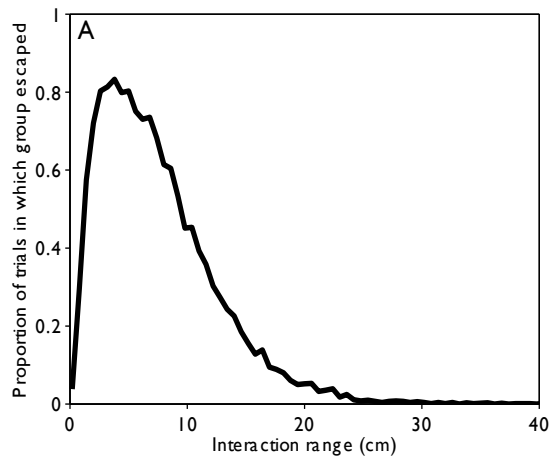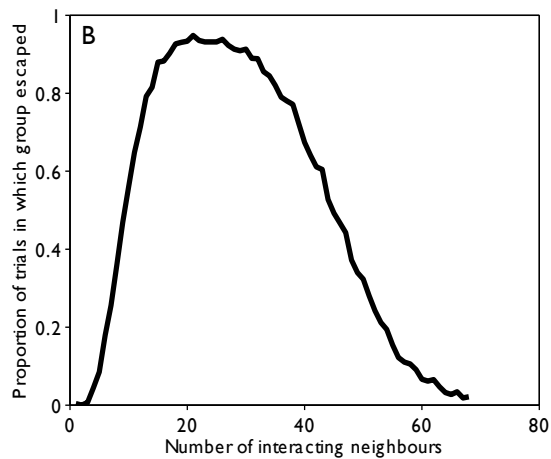

Supplement: Figure S6. How success depends on the locality of interactions. Proportion of the group that evaded the threat in model simulations where results are from 1024 simulation runs for each interaction range or number. We look at 120 particles, where 12 are initially informed. (a) Metric interactions: a  [file rsos140355supp6.pdf]

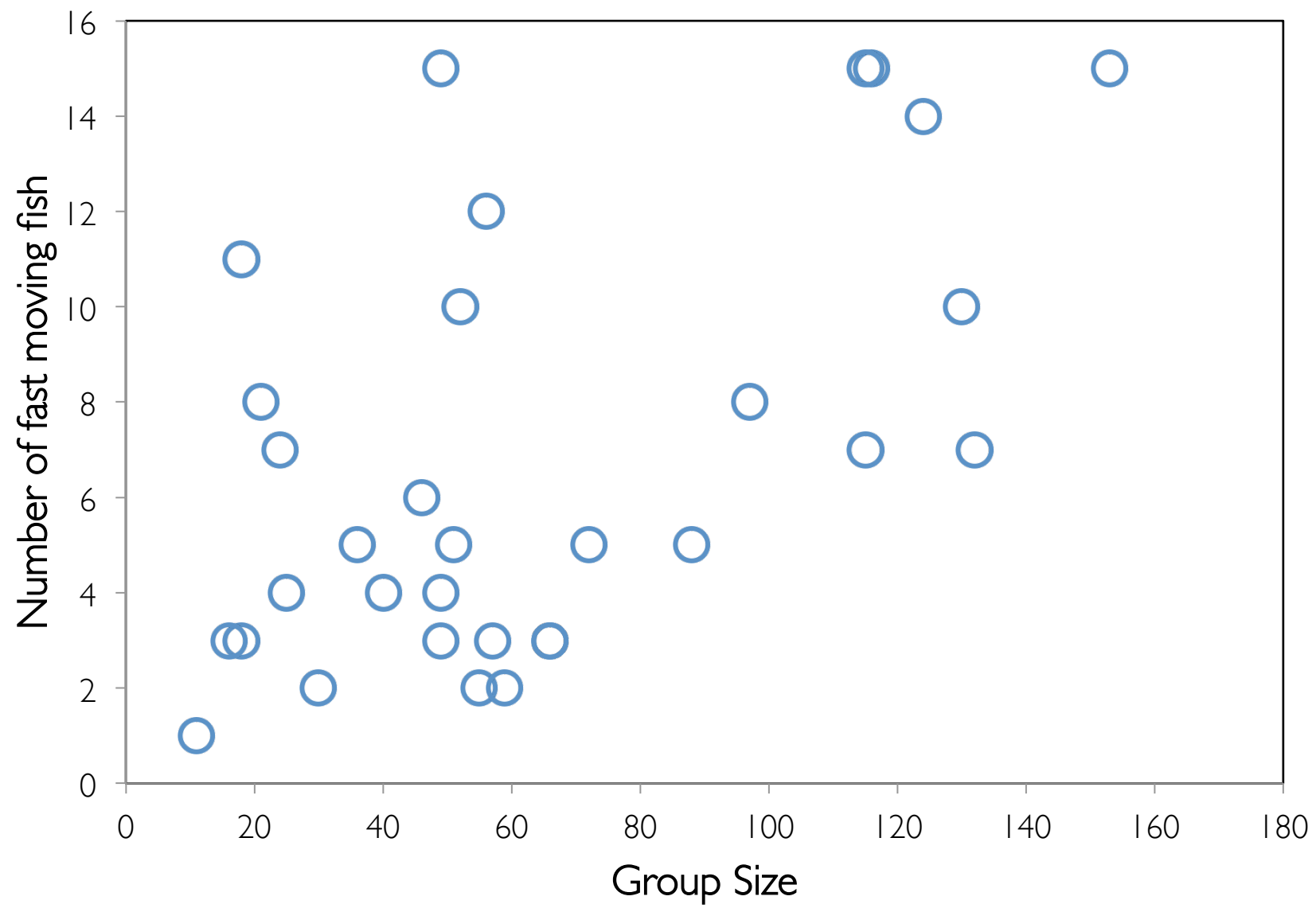

Supplement: Figure S7. The number of fast moving fishes (those individuals travelling at a speed of 0.248 m s-1 in a counter clockwise direction) within the first second following the introduction of the stimulus in a trial, plotted against the group size for that trial. [file rsos140355supp7.pdf]

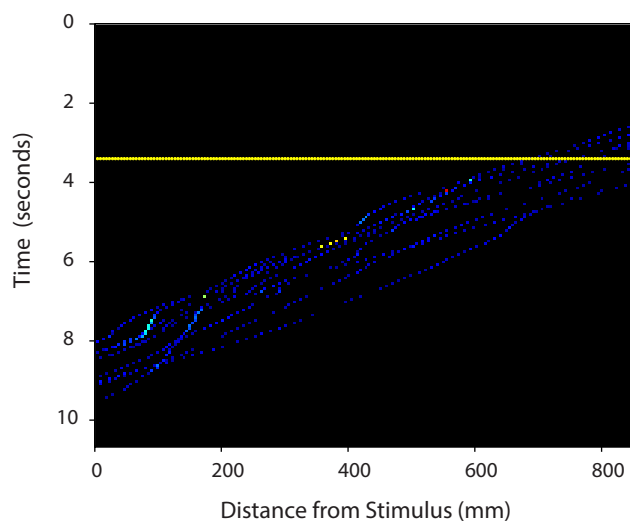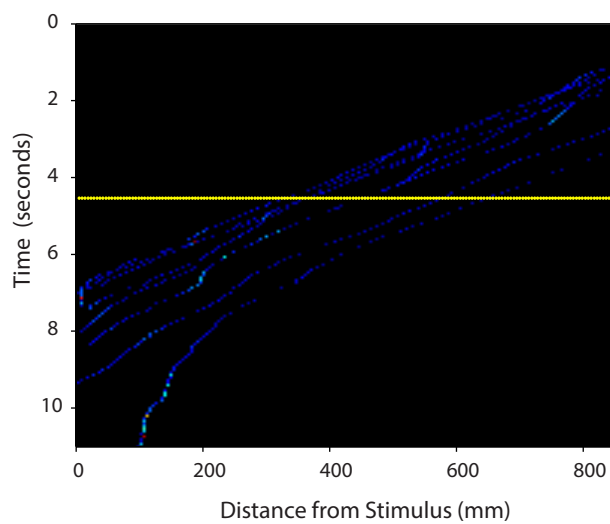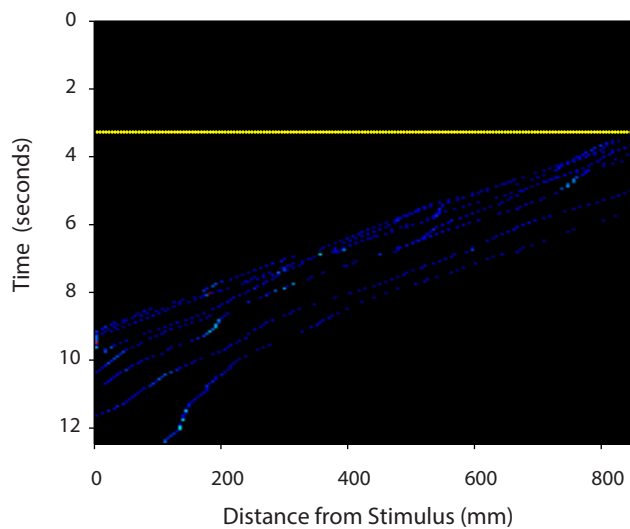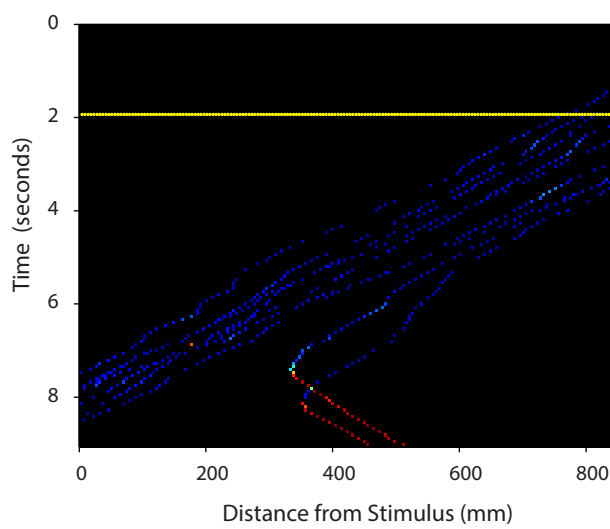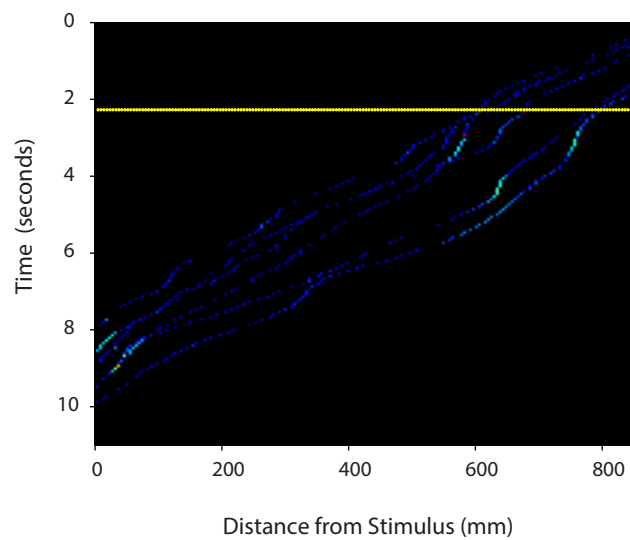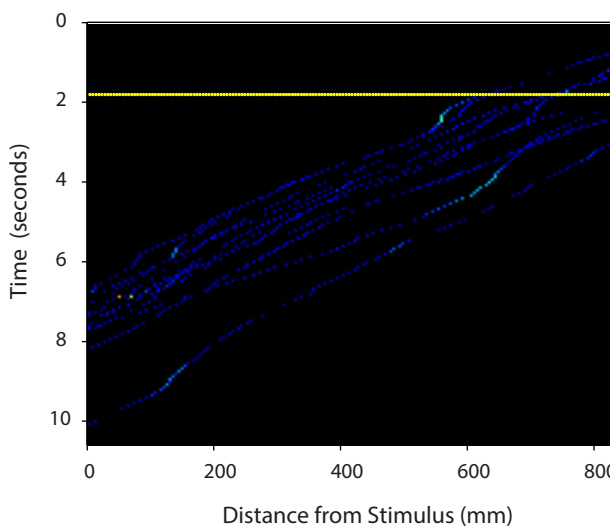

Supplement: Figure S8. Examples of fish trajectories in the control trials. The movements of fish (as in figure S2 a-d) in control trials when the stimulus was released when the nearest fishes were between 31.9 cm and 47.9 cm from the stimulus. At these distances, fish did not initiate a response to move away f [file rsos140355supp8.pdf]

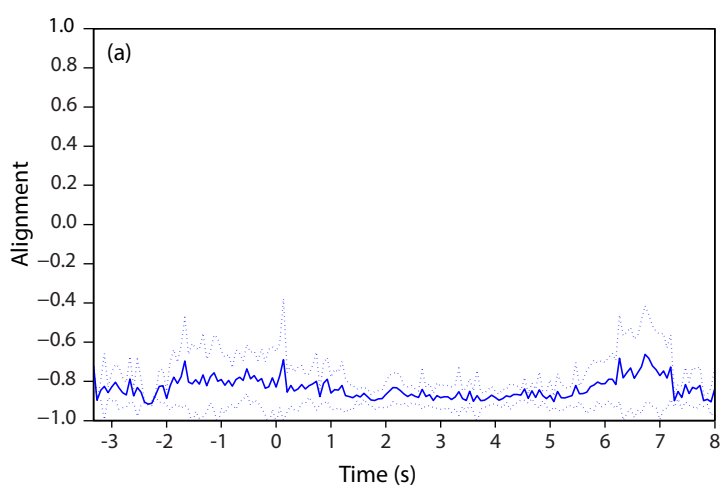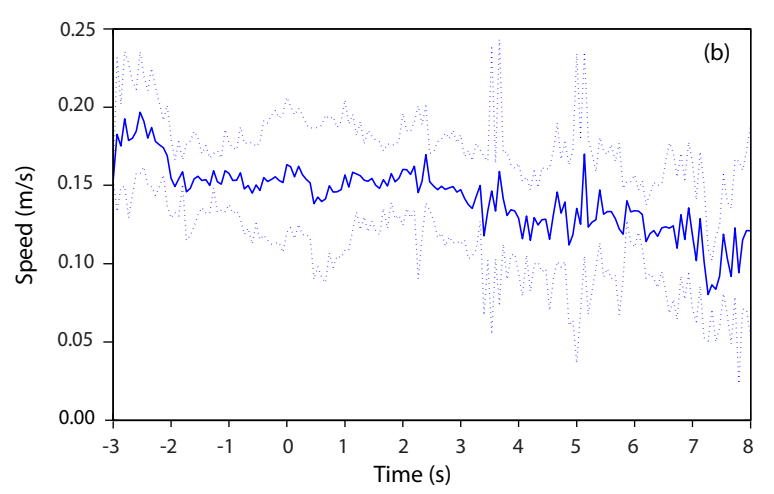

Supplement: Figure S9. Collective alignment and speed of fish in the control trials. (a) The average alignment of fish in the control trials. Unlike the other trials, fish do not show an average change in alignment, characteristic of individuals detecting the stimulus. (b) Further, they do not show any obvious  [file rsos140355supp9.pdf]

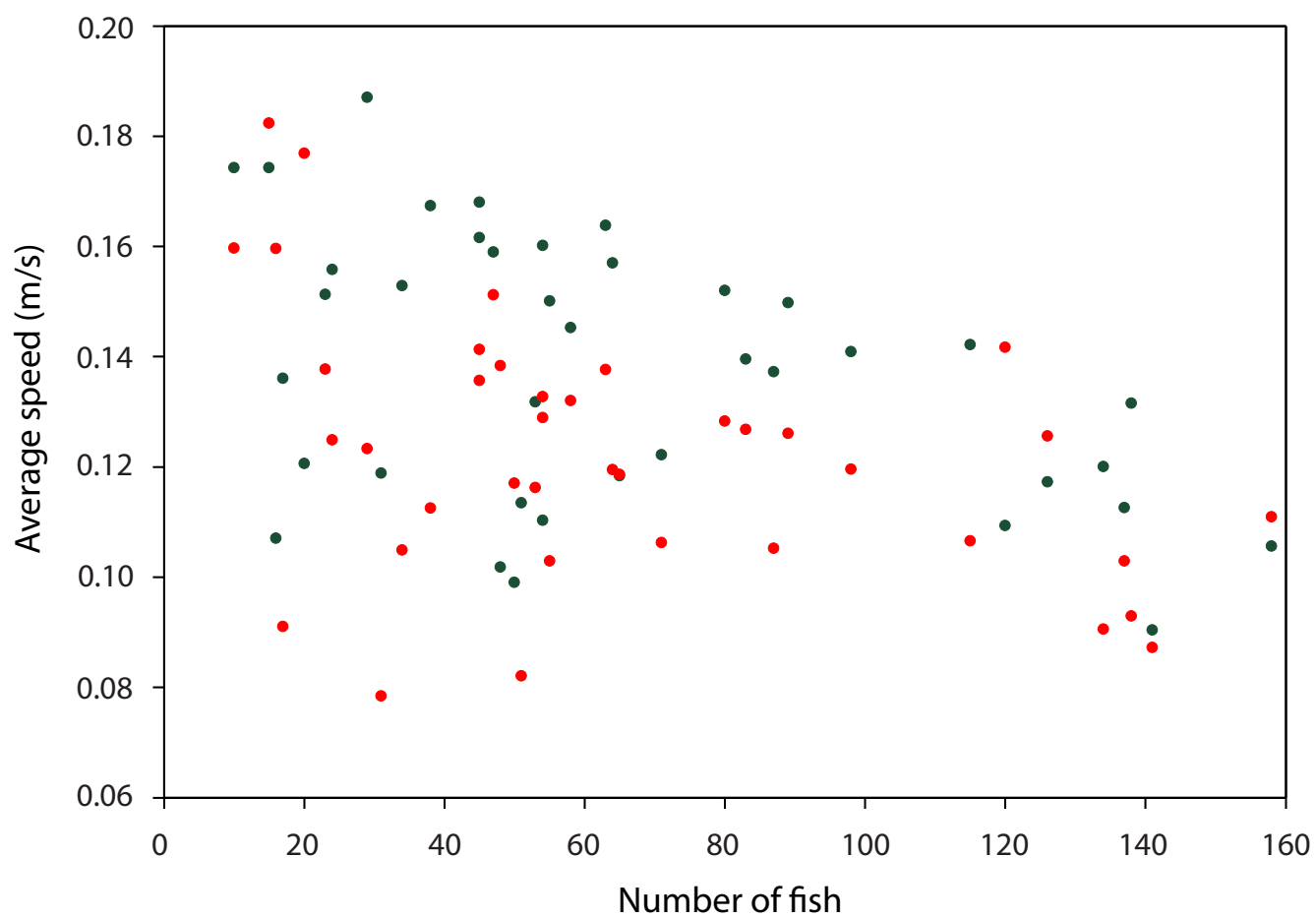

Supplement: Figure S10. Average speed of fish as a function of group size. Average speed of fish calculated during one second before (green) and after (red) the presentation of the stimulus as a function of the number of fish (each data point corresponds to one experiment). [file rsos140355supp10.pdf]
