# Supplementary material for: Initiation and spread of escape waves within animal groups
Source: R Soc Open Sci. 2015 Apr 1;2(4):140355. doi: 10.1098/rsos.140355 (PMC4448869; doi:10.1098/rsos.140355)
Supplement: Table S1. Experimental schedule. Number of fish and order of experimental and control trials (denoted by *). [file rsos140355supp11.pdf]

Day	Group size
1	51, 137,
2	54, 24, 89, 50, 17
3	71, 115, 29, 15,
4	120
5	10, 101, 83,
6	158
7	38, 20, 126,
8	138, 54, 48
9	55, 63, 34, 45,
10	141, 58
11	87, 31, 80, 53,
12	45, 23, 98, 47
13	64, 16, 134,
14	9*, 8*, 8*, 9*, 6*, 11*
15-17	Single fish experiments
