# Supplementary material for: Initiation and spread of escape waves within animal groups
Source: R Soc Open Sci. 2015 Apr 1;2(4):140355. doi: 10.1098/rsos.140355 (PMC4448869; doi:10.1098/rsos.140355)
Supplement: Supplementary material [file rsos140355supp12.pdf]

Supplementary material for: Initiation and spread of escape waves within animal groups

J.E. Herbert-Read, J. Buhl, F. Hu, A.J.W. Ward, D.J.T. Sumpter

Additional Experimental Procedures. Because we were needed to use large numbers of fish in the trials, and to comply with the reductionist policy of animal ethic protocols, we reused fish during the group experimental trials (but not the single fish experiments or control trials; see below). The same fish, however, were never used more than once per day. Further, we randomized group sizes across days (table S1). To test whether day had an effect on the behaviour of the fish, we fitted generalized linear models where day was included as a factorial variable to account for all possible variation in response. All non-significant interaction terms were removed. Whilst there was an effect of group size on the average speed of fish in the second before the stimulus entered the arena ($F_{1,25} = 14.7$, $p < 0.001$), there was no effect of day ($F_{12,25} = 1.59$, $p = 0.16$). Once again, there was an effect of group size on the speed of fish in the second after the stimulus entered the arena ($F_{1,25} = 9.74$, $p = 0.005$) but no effect of day ($F_{12,25} = 0.96$, $p = 0.51$). Finally, there was no effect of group size or day on the speed of the escape wave ($F_{1,14} = 0.13$, $p = 0.72$; $F_{12,14} = 0.94$, $p = 0.54$, respectively).

Detecting the individual that responded to the stimulus first. For each frame, we calculated the shortest Euclidian distance between the stimulus location and any fish. For each frame following the application of the stimulus, we also determined when a fish first started moving in the anticlockwise escape direction. If several fish reacted on the same frame, we considered the first reacting fish as the nearest one to the stimulus. We manually tracked the position of these first responders using a bespoke script made in MATLAB.

Detecting the fast moving fish. During the initial phase of the reaction to the stimulus, reacting fishes were usually seen accelerating to high speed. To quantify how many fish were involved in high-speed movement and what was their maximal speed, we calculated the distribution of fish speed before the stimulus (during the 30 frames immediately preceding the stimulus in all experiments, n = 34833), and determined the speed of the 99 percentile (0.253 m s^{-1}), which represented 2.03 times the average fish speed (0.124 m s^{-1} ± 5.94). We then considered a fast reacting fish as any fish that was moving anticlockwise and at a speed higher than 2 times the average initial fish speed (calculated during the first second of the experiment). We recorded the number of fast reacting fish on each frame after the reaction started (figure S4). The total number of fast reacting fish detected in each group size tested is also shown (figure S7).

Determining whether the stimulus produced a localized response. We found a strong relationship between the distance of the closest fish to stimulus and the distance from the stimulus to the first fish detected as reacting to the stimulus (r = 0.42, n = 39, p = 0.007) indicating that the first fish to switch from a clockwise (moving towards the stimulus) to an anticlockwise direction (moving away from the stimulus) was generally the closest fish to the stimulus. This supports a localised response to the stimulus. However, we also determined whether the stimulus created a localized response (and not a global response) that could not be detected by all group members using control trials. Using a different set of fish to the experimental trials, we ran control trials (n = 6) where we released the stimulus when all fish were between 31.9 cm and 47.9 cm from the stimulus (shortest Euclidean distance; tangential arc length distance is shown in figure S8). We did not re-use fish in control trials. Fish in these

trials did not initiate a response to move away from the stimulus (figure S9). Nor did they show a characteristic increase in speed or alignment following the attack (figure S9).

Effects of group size on speed. There was a strong relationship between group size and the average speed of fish before the stimulus entered the arena (r = -0.46, n = 39, p = 0.004); larger groups had slower average speeds than smaller groups (figure S10).

Interaction range and topological interactions. In order to investigate how the interaction range affected the escape wave, we first used the same parameters as above, but now varied *r*. The results of these simulations are shown in figure S6*a*. When *r* is equal to the width of the group, then the group typically adopts the direction given average direction of all individuals at start of the simulation. The average direction is given by

$$(-2.19p + (1 - p))v_{max}$$

which is positive for $p = 0.1$. Therefore, large interaction radii seldom result in direction changes in response to stimuli.

The metric interaction radii model can be replaced by a topological model where each the set of neighbours $N_i(t)$ are the set of n nearest neighbours. Now, $|N_i(t)| = n$ [52]. The probability the group changes direction as a function of *n* is shown in figure S6*b*. Again, interactions with a large number of individuals lead to adoption of the average direction of all group members and a failure to turn in response to the stimulus.

Supporting References

52. Ballerini, M., Cabibbo, N., Candelier, R., Cavagna, A., Cisbani, E., Giardina, I., Lecomte, V., Orlandi, A., Parisi, G., Procaccini, A. et al. 2008 Interaction ruling animal collective behavior depends on topological rather than metric distance: Evidence from a field study. *Proc Nat Acad Sci* 105(4), 1232-1237.

Figure S1. Collective alignment and density of fish before and after presentation of the stimulus. (*a*) Collective alignment and (*b*) density of fish before (negative seconds) and after (positive seconds) the stimulus entered the arena. The shoals show an increase in alignment and density following the entrance of the stimulus at 0 seconds. The density of fish remains high due to fish gathering on the opposite side of the arena to the stimulus.

Figure S2. Examples of the dynamics of information transfer and spatial velocity fluctuations in the experimental trials. Trials with (*a, e*) 63 fish; (*b, f*) 45 fish; (*c, g*) 98, (*d, h*) 16. (*a-d*) Each line represents a one dimensional view of the arena at a given frame where each individual's angular coordinate (in a polar coordinate system with the centre of the arena as a pole and the radius going through the stimulus position as a polar axis) is represented by a colour coded point (the colour bar indicates values in radians) representing its angle relative to the radius χ: individuals in deep blue colour are moving perpendicularly to the radius and moving in the clockwise direction, individuals in red colour are oriented perpendicular to the radius but moving anti-clockwise, and individuals in green colour are moving in a parallel direction to the radius. As the trial progresses in time (Y axis), the fish get closer to the stimulus until the stimulus enters the arena (yellow horizontal line). A clear escape response develops, with all individuals moving away from the stimulus. (*e-h*) Velocity fluctuations (see main text) measured as a function of distance during the trial. These are uncorrelated before attack, positively correlated at shorter distances and an anti-correlated at greater distances during the attack, but uncorrelated following the attack. The points at which the velocity fluctuations reach zero indicate the group's correlation length.

Figure S3. Speed profiles of individual fish reacting to the stimulus. The average speed of the first responding individual in groups (blue line) and when individuals were trialled on their own (red line) after the stimulus entered the arena at 0 seconds. Fish in both contexts show a rapid increase in speed following the attack, characteristic of fast-start responses in fish. Following the attack, they return to a swim speed similar to that of their swim speed before the stimulus. Error bars represent 1 ± SE.

Figure S4. Distribution of fish speeds before and after the presentation of the stimulus. (*a*) Mean speed of all fish in trials (± 1 SD) before (negative seconds) and after (positive seconds) the stimulus enters the arena at 0 seconds. Between -4 to 0 seconds the fish reach an average cruise speed of 0.124 m s^{-1}. Following the introduction of the stimulus, there is a general decrease in speed due to the group gathering on the opposite side of the arena to the stimulus. (*b*) The proportion of fast moving individuals (those individuals travelling the speed of 0.248 m s^{-1}) peaks at approximately 1.5 seconds following the introduction of the stimulus at 0 seconds. (*c-d*) Histogram showing the distribution of speeds of individuals in the 2 seconds before (*c*) and after (d) the stimulus had entered the arena. The long tail in (*d*) shows the speeds and relative proportions of the faster moving informed individuals.

Figure S5. The role of speed change in determining whether a group changes direction. Results of 1024 simulation runs of 80 individuals, in which 8 are informed. The dotted line shows the proportion of runs in which the group changed direction as a function of group width when informed and uninformed individuals travel at the same speed (i.e. $v_i(0) = -v_{max}$), while the solid line gives the same measure for uninformed individuals that initially travel faster than informed individuals (i.e. the standard value of $v_i(0) = -2.19v_{max}$).

Figure S6. How success depends on the locality of interactions. Proportion of the group that evaded the threat in model simulations where results are from 1024 simulation runs for each interaction range or number. We look at 120 particles, where 12 are initially informed. (*a*) Metric interactions: a particle's neighbours are all those within the interaction radius (b) Topological interactions: particles neighbours are the *k* nearest individuals. Note, these two interaction rules are largely equivalent if individuals regulate their density.

Figure S7. The number of fast moving fishes (those individuals travelling at a speed of 0.248 m s^{-1} in a counter clockwise direction) within the first second following the introduction of the stimulus in a trial, plotted against the group size for that trial.

Figure S8. Examples of fish trajectories in the control trials. The movements of fish (as in figure S2 *a-d*) in control trials when the stimulus was released when the nearest fishes were between 31.9 cm and 47.9 cm from the stimulus. At these distances, fish did not initiate a response to move away from the stimulus (average proportion of fish moving away from the stimulus in the last second of experiment: 0.068 ± 0.11).

Figure S9. Collective alignment and speed of fish in the control trials. (*a*) The average alignment of fish in the control trials. Unlike the other trials, fish do not show an average change in alignment, characteristic of individuals detecting the stimulus. (*b*) Further, they do not show any obvious changes in speed following the attack (see figure S1*a* for comparison). The gradual decrease in speed as the trial progresses is due to the fish encountering the stimulus as the trial progresses. Because the stimulus remains projected across the water, the fish slow down due to its novelty in the arena.

Figure S10. Average speed of fish as a function of group size. Average speed of fish calculated during one second before (green) and after (red) the presentation of the stimulus as a function of the number of fish (each data point corresponds to one experiment).

Table S1. Experimental schedule. Number of fish and order of experimental and control trials (denoted by *).

Movie S1. Example of one of the trials with 58 fish showing the experimental set-up. The stimulus enters the arena after 2 seconds causing the fish to turn around in an attempt to evade the stimulus.
